# Prevalence of secondhand smoke exposure at home and associated factors among middle school students in Northern Thailand

**DOI:** 10.18332/tid/117733

**Published:** 2020-02-18

**Authors:** Chakkraphan Phetphum, Narongsak Noosorn

**Affiliations:** 1 Department of Community Health, Faculty of Public Health, Naresuan University, Phitsanulok, Thailand

**Keywords:** second hand smoke, passive smoking, high school students, Thailand

## Abstract

**INTRODUCTION:**

One-third of youths in Thailand will be exposed to secondhand smoke (SHS) from family members who are smokers. This research aims to study the prevalence of and factors associated with SHS exposure at home among middle school students in Northern Thailand.

**METHODS:**

This study used a cross-sectional survey. The sample was 780 middle school students in Northern Thailand. A self-administered questionnaire was used for data collection and analyses were performed using a chi-squared test and multiple logistic regression.

**RESULTS:**

Of the respondents, 46.8% reported that they had been exposed to SHS at home. The main SHS sources were from fathers (45.4%), relatives (24.1%), siblings (12.4%), mothers (3.3%), and neighbours and guests (14.8%). The factors associated with SHS exposure at home included: household members who were smokers (OR=7.43; 95% CI: 5.17–10.68; p<0.001), home without a smoke-free rule (OR=3.40; 95% CI: 1.85–6.24; p<0.001), household members who were alcohol drinkers (OR=2.29; 95% CI: 1.59–3.30; p<0.001), and living in homes with ≤3 rooms (OR=1.79; 95% CI: 1.21–2.63; p=0.003).

**CONCLUSIONS:**

Thai student’s exposure to SHS at home is high, especially when household members smoke and they live in a home without a smoke-free rule. Our findings highlight the need for policies and interventions to establish smoke-free homes.

## INTRODUCTION

Secondhand smoke (SHS) comes from burning tobacco products and exhaled by smokers near non-smokers. There is no risk-free level of SHS exposure; even brief exposure can be harmful to health^1^. SHS has both short-term and long-term impacts, such as eye irritation, headache, cough, sore throat, dizziness and nausea, and maximizes the risk of heart disease, stroke and lung cancer^2^. Each year, SHS exposure is the cause of about 0.6 million deaths worldwide and results in 10.9 million disability adjusted life years (DALYs)^3^. Children and youths are particularly vulnerable to SHS due to higher breath frequency and lower ability to handle serious adverse health effects because of immature liver metabolism and other clearing mechanisms^4-6^. The World Health Organisation (WHO) estimated that half of the children and youths worldwide will be exposed to the effects of SHS from family members who smoke^7^. Similarly, 57.0% of Asian children and youths are exposed regularly to SHS in their homes^8^.

Thailand implemented control laws to limit smoking and tobacco product use since 1992, revised in 2017. Under these laws, smoking is prohibited in indoor public places, workplaces, public vehicles, bus stops, and gas stations. While workplaces and public smoking bans have proven to be successful in reducing exposure to SHS in public areas, from 49.6% in 2011 to 25.5% in 2017^9^, private areas cannot be directly targeted by smoke-free legislation. Evidence shows that smoking bans in public places lead to more smoking in the home, which increases SHS exposure of non-smokers, especially children, by family members^10^. This phenomenon is called the ‘displacement effect of smoke-free legislation’^11^. A recent study showed that nearly 35% of children are exposed to SHS at home in Thailand^12^. The results from national surveys show that the main SHS source at home is the father’s smoking, with two-thirds (68.2%) smoking mostly in front of a fence, on the terrace, or in a rest room^13^.

Recently, a number of studies showed that the characteristics of the home members, and of the home itself, affect non-smoking youths’ exposure to SHS. Those who live in multi-unit housing and small-size buildings with fewer accommodation rooms are more likely to be exposed to SHS at home^14-20^. Youths who live in homes with fewer children and youths, have a lower household income, or their father and/ or mother smoke, have a higher probability of being exposed to SHS at home^21-25^. Furthermore, restriction of smoking at home, by the agreement of the house members, helps to minimize exposure to SHS of household members and children^19,26,27^.

Exposure to SHS among children and youths could become a major issue in Thailand due to implementation of anti-smoking legislation and lack of provision to restrict smoking at home. Therefore, we need to understand both the level of SHS exposure in this population and its determinants, so that appropriate preventative measures can be taken. The aim of this study was to determine the prevalence of SHS exposure at home and the factors associated with it, among middle school students in Northern Thailand.

## METHODS

A cross-sectional survey was conducted in 5 provinces of Northern Thailand (Phitsanulok, Tak, Petchabun, Sukhothai, and Uttaradit). The sample group was 788 middle school students (Grade 8) from the 5 provinces. We calculated the sample size with the finite proportional population for the prevalence of SHS exposure at home among youth; based on a p=0.33812, an error (delta) of 10.0% of p (delta=0.0338), and a confidence level of 95% (α=0.05), to obtain 743 students.

Participants were selected by a combination of stratified sampling with probability proportional to size, and classroom survey technique. First, all the middle schools of the study area were grouped in 5 strata (5 provinces) according to their population. Then, one school from each stratum was selected with probability proportional to size and one class room from each school was selected with classroom survey technique. The total sample obtained from the classroom survey technique was 788 persons, which was more than the required sample size by 45 persons.

Approval for this study was obtained from the human ethics research committee, Naresuan University (No. IRB 0109/61), and consent was from the students’ parents. Participation in the survey was voluntary, and the student participation rate was 100%. The questionnaire was distributed to the participants to complete within 90 minutes and returned directly to the researchers on the same day. However, because 8 of the questionnaires were less than 80% completed, they were excluded and only 780 were used in the analysis. Data were collected by trained data collectors during January and February 2018. Students completed a self-administered questionnaire containing 8 questions for the participants who were not exposed to SHS at home and 11 questions for those who were exposed. All questions had an Index of Item Objective (IOC) value greater than 0.5.

### Dependent variables

SHS exposure at home was the dependent variable of the study. It was defined by the question: ‘Have you been exposed to SHS at home within the previous 3 months?’, with response option ‘Yes/No’. When a participant indicated that they had experienced SHS exposure at home within the previous 3 months, they were asked about the frequency of SHS exposure at home (number of times), the time of SHS exposure at home (06.00–08.00 am or 05.00–08.00 pm), the location where SHS exposure occurred at home (balcony, backyard, common room, kitchen, bedroom, toilet, or other location), and the source of SHS (father, mother, relatives, siblings, neighbours, and guests).

### Independent variables

#### Sociodemographic factors

The self-reported sociodemographic factors investigated were: sex, age, household income, whether they lived with household members who were smokers, whether they lived with household members who were alcohol drinkers, and whether they lived in a home without a smoke-free rule, this included homes with or without smokers, and whether smokers were not allowed to smoke inside the home.

#### Building environment factors

The self-reported building environmental factors investigated were the student’s type of housing (single house vs multi-unit housing, i.e. condominium, flat, commercial building or multi-family dormitory) and number of rooms in the student’s house.

### Statistical analysis

The data gathered were analyzed through the statistical software IBM SPSS Statistics version 17 for Windows. First, frequency runs were explored to present descriptive information about the sample (including percentages and means). Cross tabulations between each dependent variable and all independent variables were performed to explore the associations between SHS exposure at home and nominal or ordinal scaled independent variables (i.e. gender, type of house, living with household members who were smokers, living with household members who were alcohol drinkers, and living in a home without a smoke-free rule). The continuous variables were dichotomised by using the mean (i.e. age ≤14 or >14 years), parents’ income (≤12547 or >12547 THB, Thai Baht) and number of rooms in the house (≤3 or >3 rooms). We used the chi-squared test of independence to analyze the associations of two variables with multiple categories. Bivariable analyses were conducted using crude odds ratios (ORs) to highlight the associations between SHS exposure at home and all independent variables (p<0.25) and we used a multiple logistic regression method for control of the influence of the related variables by using adjusted odds ratios (AOR). A two-sided p<0.05 was considered statistically significant.

## RESULTS

Most of the sample was female (53.1%), aged 13–14 years (65.9%), living in a single house (78.1%), and the house had ≤3 rooms (71.4%). Most lived with a household member who was a smoker (57.9%), lived with a household member who was an alcohol drinker (59.3%), and living in a home without a smoke-free rule (88.5%).

Almost half of the participants (46.8%) had been exposed to SHS at home. They were exposed to SHS at home 1–2 times per day (72.0%), during the period after school or during the evening 5.00–8.00 pm (80.2%), the main source of SHS at home was the father (45.4%), followed by relatives (24.1%), siblings (12.4%), mother (3.3%) and neighbours and guests (14.8%). The locations that most students were exposed to SHS at home were outside the house such as the balcony or backyard (53.5%), common room (21.3%), toilet (11.1%), kitchen (7.8%), and bedroom (6.3%) (Table 1).

**Table 1:** Sample characteristics and SHS exposure at home, Northern Thailand, 2018 (N=780 )

*Variables*	*Total*		*Exposed*		*Not exposed*		*χ2*	*p*

	*n*	*%*	*n*	*%*	*n*	*%*		
**Sex**								
Female	366	46.9	175	47.8	191	52.2	0.29	0.592
Male	414	53.1	190	45.9	224	54.1		
**Age** (years)								
≤14	514	65.9	240	46.7	274	53.3	0.01	0.937
>14	266	34.1	125	47.0	141	53.0		
**Type of home**								
Single house	609	78.1	298	48.9	311	51.1	5.10	0.024*
Multi-unit housing	171	21.9	67	39.2	104	60.8		
**Number of rooms**								
>3	557	71.4	284	51.0	273	49.0	13.76	<0.001*
≤3	223	28.6	81	36.3	142	63.7		
**Household income** (THB)^a^								
>12547	561	71.9	279	49.7	282	50.3	6.31	0.012*
≤12547	219	27.2	86	39.6	133	60.4		
**Household smoker**(s)								
No	431	57.9	300	69.6	131	30.4	201.31	<0.001*
Yes	349	42.1	65	18.6	284	81.4		
**Household alcohol drinker**(s)								
No	452	55.3	272	60.2	180	39.8	77.31	<0.001*
Yes	328	44.7	93	28.4	235	71.6		
**Home without smoke-free rule**								
Yes	90	11.5	19	21.1	71	78.9	26.96	<0.001*
No	690	88.5	346	50.1	344	49.9		

a THB: Thai baht 1000 about US$33.

* p<0.05

The association between the independent variables and SHS exposure at home is shown in Table 1. The sociodemographic factors that were significantly associated with SHS exposure at home included: lower household income (χ^2^=6.31; p=0.012), living with a household member who was a smoker (χ^2^=201.31; p<0.01), living with a household member who was an alcohol drinker (χ^2^=77.31; p<0.01), and living in a home without a smoke-free rule (χ^2^=26.96; p<0.01). The building environment factors that were significantly associated with SHS exposure at home included: living in a single house (χ^2^=5.10; p=0.024) and the house having ≤3 rooms (χ^2^=13.76; p<0.01). However, the prevalence of exposure to SHS did not differ significantly by age or sex of the students (Table 1).

The bivariate and multivariate logistic regression models of exposure to SHS at home are shown in Table 2. In the bivariate analysis, all independent variables comprised the factors associated with SHS exposure at home (p<0.25). The students who were male (OR=1.18; 95% CI: 0.97–1.43; p=0.095) and aged ≤14 years (OR=1.14; 95% CI: 0.96–1.36; p=0.134) were more likely to report exposure to SHS at home than those who were female and aged >14 years. The students who lived in a single house (OR=1.55; 95% CI: 1.14–2.11; p=0.005), that had ≤3 rooms (OR=1.75; 95% CI: 1.33–2.30; p<0.001), and had a household income ≤12547 THB (OR=1.52; 95% CI: 1.16–2.01; p=0.003) were more likely to report SHS exposure at home than those who lived in multi-unit housing, a house with >3 rooms, and a household income >12547 THB. Moreover, the students who lived with a household member who was a smoker (OR=4.37; 95% CI: 3.34–5.72; p<0.01), who was an alcohol drinker (OR=2.53; 95% CI: 1.99–3.21; p<0.01), and lived in a home without a smoke-free rule (OR=3.74; 95% CI: 2.25–6.20; p<0.001) were more likely to report SHS exposure at home than those who lived with household members who were not smokers, not alcohol drinkers, and lived in a home with a smoke-free rule.

**Table 2:** Binary and multiple logistic regression models for factors associated with SHS exposure at home, Northern Thailand, 2018 (N=780 )

*Variables*	*Crude odds ratio*			*Adjusted odds ratio^b^*		

	*OR*	*95% CI*	*p*	*OR*	*95% CI*	*p*
**Sex**						
Female	1					
Male	1.18	0.97–1.43	0.095*	1.30	0.92–1.83	0.141
**Age** (years)						
≤14	1					
>14	1.14	0.96–1.36	0.134*	0.99	0.69–1.42	0.951
**Type of home**						
Single house	1					
Multi-unit housing	1.55	1.14–2.11	0.005*	0.88	0.58–1.33	0.539
**Number of rooms**						
>3	1					
≤3	1.75	1.33–2.30	<0.001*	1.79	1.21–2.63	0.003**
**Household income** (THB)^a^						
>12547	1					
≤12547	1.52	1.16–2.01	0.003*	1.28	0.86–1.89	0.224
**Household smoker**(s)						
No	1					
Yes	4.37	3.34–5.72	<0.001*	7.43	5.17–10.68	<0.001**
**Household alcohol drinker**(s)						
No	1					
Yes	2.53	1.99–3.21	<0.001*	2.29	1.59–3.30	<0.001**
**Home without smoke-free rule**						
Yes	1					
No	3.74	2.25–6.20	<0.01*	3.40	1.85–6.24	<0.001**

a THB: Thai baht 1000 about US$33.

b b Adjusted for all variables listed.

* p<0.25.

** p<0.05.

The final multivariate logistic regression model was fit for the proportional odds assumption (p=0.892). In the multivariate analysis, the students who lived with a household member who was a smoker had odds of being exposed to SHS at home 7.43 times that of those of who lived in a home without a smoker (OR=7.43; 95% CI: 5.17–10.68; p<0.001); the students who lived in a home without a smoke-free rule had odds of being exposed to SHS at home 3.4 times that of those who lived in a home with a smoke-free rule (OR=3.40; 95% CI: 1.85–6.24; p<0.001); the students who lived with a household member who was an alcohol drinker had odds of being exposed to SHS at home 2.29 times that of those who lived in homes without an alcohol drinker (OR=2.29; 95% CI: 1.59–3.30; p<0.001). Finally, students whose house had ≤3 rooms had odds of being exposed to SHS at home 1.79 times that of those who lived in a home that had >3 rooms (OR=1.79; 95% CI: 1.21–2.63; p=0.003). The other variables were not significantly associated with exposure to SHS at home (Table 2.)

## DISCUSSION

This study found that the prevalence of SHS exposure at home was 46.8%, higher than the prevalence of SHS exposure at home of children and youths in 2015 (35.0%)^12^. This also supports the possibility of a displacement effect of smoke-free legislation^10,11^. Although they were exposed to SHS at home 1–2 times per day, it is enough to have negative effects on health^1^. The period after school was the time they were exposed to SHS the most, as it was the period when they spent time with family and household members, chatting, watching TV, or having dinner. The students were exposed to secondhand smoke outside the house building the most. In line with published studies, the source of SHS exposure at home was mostly from the household members, especially from their father, while the locations where the students were exposed to SHS at home the most were outside the house building such as the balcony or the backyard^13^. There was a high probability that the smoke was dispersed to house members who were inside the house because Thai-style houses apply the natural ventilation system such as opening the door wider for air flow. This was in line with the research results conducted in South Korea where the natural air ventilation system posed a higher risk of exposure to secondhand smoke than the closed-system, or air conditioning system, with 1.27 times odds (OR=1.27; 95% CI: 1.01–1.60; p=0.038)^20^.

The multivariate analysis results, in terms of the characteristics of the house, illustrated that the students who lived in a house with 1–3 rooms were likely to be exposed to secondhand smoke 1.785 times that of those who lived in a house that had >3 rooms, consistent with previous research^28^. It is possible that the smaller number of rooms in the house had fewer panels to mitigate the dispersion of secondhand smoke. Moreover, it was the limitation of space that students had less chance to avoid secondhand smoke exposure in the house^29^. Meanwhile, the three sociodemographic factors affected the students’ exposure to secondhand smoke, and the coexistence with at least one smoker in the house increased the possibility of exposure to secondhand smoke 7.43 times that of students who were living in a house without a smoker, in line with previous research^30^. This was reasonable because as long as there was a source of smoke in the house, it was hard to prevent non-smokers from being exposed to secondhand smoke. Therefore, the best solution is to make the family members quit smoking^31^. A house member who drank alcohol, was a new variable that no one had yet researched on. However, we were interested to do so because Thai people who drink alcohol normally smoke. In cases where a person did not smoke, at least one of the guests they invited to drink at their home smoked, which increased the chance of secondhand smoke exposure of house members. This result confirms that students who live in a house where at least one member drinks alcohol has higher odds of exposure to secondhand smoke by 2.29 times that of those living in a house without an alcohol drinker. The students who lived in a home without a smoke-free rule had 3.40 times the odds of exposure to secondhand smoke. This variable was based on the restriction of smoking in areas agreed by the house members to prevent children or non-smokers of the family being exposed to secondhand smoke. This measure has become more popular in the UK during the past 20 years^26^ because there are research results that show that such a rule or agreement mitigated the odds of secondhand smoke exposure^18,27^ and helped smokers to reduce their amount of cigarettes per day and finally to quit^32^. Besides, the smoking-free rule also minimized the chance of smoking among the youths who were house members, or at least it helped to extend or prolong their smoking trial period^33^.

### Limitations

The interpretation of this study’s results has some limitations. First, this was a cross-sectional survey. Therefore, only associations and not causalities can be drawn. Second, we used self-report SHS exposure experienced by the middle school students within the previous 3 months and measured the SHS exposure based on the detection of SHS by smell without biochemical verification. Therefore, possible recall bias may have resulted in under-reporting, such as receiving less or light smoke, no strong smell or good scent, or getting used to the smoke smell. Further study is needed using more specific SHS markers to provide a better understanding of SHS exposure at home, such as cotinine levels in non-smoking students^32^. Future studies should have a larger sample and number of strata, and the design effect should be applied to address the issue of the cluster effect of respondents from the same classroom.

## CONCLUSIONS

The study results show that nearly half of middle school students are exposed to SHS at home, in Northern Thailand. SHS exposure at home was associated with living with a household member who was a smoker, living in a home without a smoke-free rule, living with a household member who was an alcohol drinker, and living in a house with few rooms. Our findings highlight the need for effective prevention measures to protect students from SHS exposure at home by supporting the education of the family on the health risks of SHS and encouraging the family members who smoke to quit early, avoid smoking at home, and to implement a smoke-free home rule.
